# The Consolidation of Implicit Sequence Memory in Obstructive Sleep Apnea

**DOI:** 10.1371/journal.pone.0109010

**Published:** 2014-10-15

**Authors:** Eszter Csabi, Maria Varszegi-Schulz, Karolina Janacsek, Nick Malecek, Dezso Nemeth

**Affiliations:** 1 Institute of Psychology, University of Szeged, Szeged, Hungary; 2 Somnocenter, Szeged, Hungary; 3 Department of Clinical Psychology and Addiction, Eotvos Lorand University, Budapest, Hungary; 4 Imaging Research Center, University of Texas, Austin, Texas, United States of America; Utrecht University, Netherlands

## Abstract

Obstructive Sleep Apnea (OSA) Syndrome is a relatively frequent sleep disorder characterized by disrupted sleep patterns. It is a well-established fact that sleep has beneficial effect on memory consolidation by enhancing neural plasticity. Implicit sequence learning is a prominent component of skill learning. However, the formation and consolidation of this fundamental learning mechanism remains poorly understood in OSA. In the present study we examined the consolidation of different aspects of implicit sequence learning in patients with OSA. We used the Alternating Serial Reaction Time task to measure *general skill learning* and *sequence-specific learning*. There were two sessions: a learning phase and a testing phase, separated by a 10-hour offline period with sleep. Our data showed differences in offline changes of general skill learning between the OSA and control group. The control group demonstrated offline improvement from evening to morning, while the OSA group did not. In contrast, we did not observe differences between the groups in offline changes in sequence-specific learning. Our findings suggest that disrupted sleep in OSA differently affects neural circuits involved in the consolidation of sequence learning.

## Introduction

Currently, there is a growing interest within cognitive neuroscience and neuropsychology to understand the underlying mechanisms of memory consolidation; namely, how newly acquired and initially labile memory representations become stabile and resistant to interference and forgetting [1]. Consolidation can be observed as no deterioration of the previously acquired knowledge over the offline period, nevertheless in some cases even offline enhancement can occur. Many studies indicate that sleep contributes to the consolidation of memory traces by enhancing neuronal plasticity [2]–[6]. Sleep-related enhancement in declarative memory is clearly demonstrated [7]–[9], but the beneficial effect of sleep on the consolidation of non-declarative (i.e. procedural) knowledge is still controversial. Previous studies that focused on healthy populations found greater improvement in a procedural sequence learning task after a period of sleep than after an equivalent time of wakefulness [10], [11]. By contrast, several recent studies failed to find sleep-related improvement in sequence learning [12]–[15]. The controversial results might be explained by task complexity, for example varying in sequence length and structure. Moreover, some sequence learning tasks used in these studies were unable to separate two aspects of sequence learning, namely general practice-dependent speed-up (so called general skill learning) and sequence-specific learning [10], [11], [16]. In the present study, we used the Alternating Serial Reaction Time (ASRT) task [17] to extend previous research by separating and measuring both general skill learning and sequence-specific learning. In this task some runs of three consecutive stimuli (triplets) are more frequent than others. With practice people become faster in responding to these high frequency triplets compared to the low frequency ones, revealing sequence-specific aspects of learning. In contrast, a general speed-up irrespectively of the triplet frequencies is considered to be a result of the general skill aspect of learning in this task [12], [14].

Previous studies suggest that sleep disorders (e.g., insomnia) lead to weaker consolidation both of declarative and non-declarative memory [18], [19]. One of the most frequent sleep disorders is obstructive sleep apnea (OSA) which is characterized by repeated episodes of upper airway obstruction during sleep, resulting in hypoxia, which leads to repetitive arousals from sleep disturbing normal sleep patterns [20]. Deficits in working memory [21], [22], attention, executive functions [23]–[26], short and long-term verbal and visual memory have been demonstrated in OSA [25], [27], [28] indicating structural changes in brain circuits crucial for memory [29]. Nevertheless, sequence learning has not been extensively characterized in OSA. Lojander, Kajaste, Maasilta & Partinen [30] have found poor performance in sequence learning in patients with apnea. In contrast, other studies showed intact performance on a less complex, deterministic sequence learning task [31] and also on a more complex, probabilistic sequence learning task [22], but they found weaker word recall and working memory performance, respectively.

The aforementioned studies investigated on the effect of sleep disorders on learning and memory functions in general but not on the overnight consolidation of the acquired knowledge. Focusing on consolidation, Kloepfer and colleagues [32] examined the memory performance before and after sleep in moderate OSA. They revealed that OSA patients showed reduced declarative (verbal) and non-declarative memory performance after sleep compared to healthy control participants. It is important to note that this study measured non-declarative memory by a motor adaptation task and not by a sequence learning task. To our knowledge, only one study focused on the consolidation of sequence learning in OSA and demonstrated that OSA can negatively affect memory consolidation on a relatively simple motor sequence learning task [16]. Nevertheless, this study used an explicit sequence learning task (fingertapping) with deterministic sequence structures. The aim of the present study was to go beyond previous research in three ways:

1. investigating the consolidation processes in OSA by a more complex sequence learning task, namely the sequence structure is not deterministic but probabilistic;
2. we use an implicit sequence learning task and not explicit (for example [16]),
3. the task used here enables us to separately analyze the consolidation of two aspects of sequence learning, namely general skill and sequence-specific learning.

Based on the previous sleep studies that used implicit probabilistic sequence learning tasks [12], [14], our hypothesis is that OSA participants will not show deterioration in sequence-specific and general skill learning over the offline period.

## Methods

### Participants

Seventeen newly diagnosed, untreated patients with OSA participated in the experiment (average age: 52.41 years, SD: 9.67; average education: 12.65 years, SD: 2.18; 2 females/15 males). OSA was diagnosed by a board-certified sleep-physician based on a full night of clinical polysomnography. The mean Apnea-Hypopnea Index (AHI) was 53.05 events/hour (SD: 23.26 (Range: 21.1–117.3). Pathological level of AHI was defined as 15 or more per hour [20]. The mean total sleep time (TST) was 330.52 mins (SD: 48.65). Aside from OSA, participants did not suffer from any developmental, psychiatric or neurological disorders as established in a full neurological exam by a board-certified neurologist.

The control group consisted of seventeen healthy participants and was matched by age (average age: 54.24 years, SD: 7.29) and by working memory performance. Working memory capacity was assessed by two widely-used neuropsychological tests: the Backward Digit Span Task (BDST) [33], [34] and Listening Span Task (LST) [35], [36]. There were no significant differences between the two groups in these tasks (BDST: t(32) = 1.116, *p* = 0.27, LST: *t*(32) = 0.170, *p* = 0.87). These criteria were included to eliminate the effect of working memory, as previous studies in healthy participants revealed a relationship between working memory and implicit sequence learning [37], [38]. However there is also evidence that the two systems are independent of each other [39]–[41] (for review see Janacsek & Nemeth [42]). Control participants did not suffer from any developmental, psychiatric or neurological disorders and did not have sleeping disorders. All participants provided signed informed consent and received no financial compensation for their participation. Ethics approval was obtained by the Psychology Ethical Committee at the University of Szeged, Institute of Psychology.

### Procedure

There were two sessions in the experiment: a Learning Phase (Session 1) and a Testing Phase (Session 2) for both the OSA and the healthy control group. The sequence learning performance was assessed between 7 and 8 PM prior to sleep (Learning Phase) and between 7 and 8 AM after sleep (Testing Phase), thus the average interval between the Learning and Testing Phase was 12 hours. Between the two sessions AHI was measured in a full night of polysomnography in SomnoCenter's sleep lab (Szeged, Hungary). During the data collection, subjects' caffeine and nicotine intake was restricted.

### Alternating Serial Reaction Time (ASRT) Task

We used the modified version of the ASRT task in which a stimulus (a picture of a dog's head) appeared in one of four empty circles on the screen [12]. Before beginning the task, detailed instructions were read to participants. They were instructed to press the button corresponding to the stimulus location as quickly and as accurately as possible [12]. The computer was equipped with a special keyboard with four marked keys (Y, C, B and M on a QWERTZ keyboard; thus, compared to the English keyboard layout, the location of the buttons Z and Y were switched), each corresponding to one of the horizontally aligned circles. Session 1 (Learning Phase) consisted of 25 blocks, with 85 key presses in each block – the first five stimuli were random for practice purposes, then an eight-element alternating sequence (e.g., 2r1r4r3r, where numbers represent the four places on the screen, and r represents an event randomly selected from the four possible places) repeated ten times. Similarly to earlier studies [12], stimuli were presented 120-ms after the previous response (response-to-stimulus interval, RSI). Each block required about 1.5 minutes and the entire session took approximately 30–40 minutes. Between blocks, participants received feedback about their overall reaction time and accuracy on the screen and then rested 10 to 20 seconds before starting a new block. Session 2 (Testing Phase) consisted of 5 blocks; the number of key presses and the RSI were the same as in Session 1 and this Testing Phase took approximately 5–10 minutes to complete.

A different ASRT sequence was selected for each participant based on a permutation rule such that each of the six unique permutations of the 4 repeating events occurred. Consequently, six different sequences were used across participants [12].

As there is a fixed sequence in the ASRT alternating with random stimuli (e.g., 2r1r4r3r), some triplets or runs of three consecutive stimuli occur more frequently than others. For example, 2_**1**, 1_**4**, 4_**3**, and 3_**2** occur more often because the third element (bold numbers) can be derived from the sequence or can also be a random element (if the sequence is 2r1r4r3r). In contrast, 1_**2** or 4_**1** occur less often because the third element can only be random. Following previous studies [12], [14], we refer to the former as high-frequency triplets and the latter as low-frequency triplets. Out of the 64 possible triplets, each 16 high frequency triplets occur on approximately 4% of the trials, about 5 times more often than the low-frequency triplets. Note that the final event of high-frequency triplets is therefore more predictable from the initial event compared to the low-frequency triplets (also known as non-adjacent second-order dependency, see in Remillard [43]).

Previous studies have shown that as people practice the ASRT task, they come to respond more quickly to the high-frequency triplets than low-frequency triplets, revealing sequence-specific learning [14], [44]. In addition, general skill learning is revealed by the overall speed-up during the practice, irrespectively of the triplet types. Thus, we are able to measure both sequence-specific and general skill learning in the ASRT task.

To explore how much explicit knowledge participants acquired about the task, we administered a short questionnaire (previously used in Song and colleagues [12], Nemeth and colleagues [14]) after the task. This questionnaire included increasingly specific questions such as “Have you noticed anything special regarding the task? Have you noticed some regularity in the sequence of stimuli?” The experimenter rated subjects' answers on a 5-item scale, where 1 was “Nothing noticed” and 5 was “Total awareness”. None of the participants in either the OSA or control group reported noticing the sequence in the task.

### Statistical analysis

To facilitate data processing, the blocks of ASRT were organized into epochs of five blocks. The first epoch contains blocks 1–5, the second epoch contains blocks 6–10, etc. Participants' accuracy remained very high throughout the test (average >96% for both groups), therefore we focused on reaction time (RT) for the analyses reported. We calculated RT medians for correct responses only (following the standard protocol, see in [12], [14], [17], [44]), separately for high- and low-frequency triplets and for each participant and each epoch. Note that for each response (n), we defined whether it was a high- or a low-frequency triplet by considering whether it is more or less predictable from the event n-2. For the analyses reported below, as in previous research [12], [14], two kinds of low-frequency triplets were eliminated: repetitions (e.g., 222, 333) and trills (e.g., 212, 343). Repetitions and trills were low frequency for all participants and people often show pre-existing response tendencies to them [44]. So by eliminating them we attempted to ensure that any high- versus low-frequency differences were due to learning and not to pre-existing tendencies.

## Results

### Online learning during Session 1 (Learning Phase)

**Figure 1:**
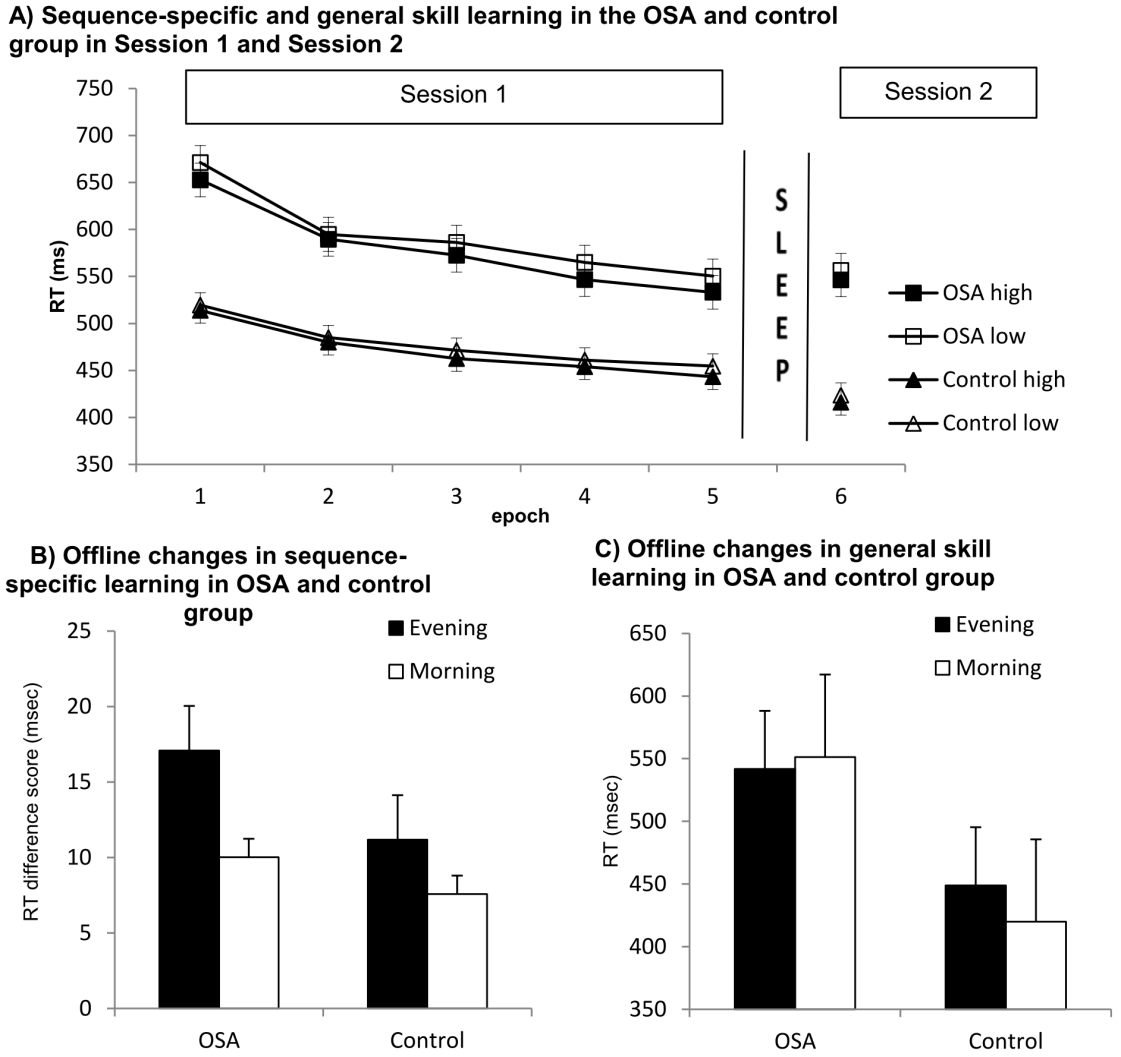
Results of sequence learning and consolidation in the OSA and control group. **A**) Results of sequence-specific and general skill learning in OSA and control group in Session 1 and Session 2: Although the OSA group was generally slower in Session 1, both groups showed significant sequence-specific and general skill learning. There were no differences in learning between the groups; the pattern of learning was similar in the OSA and control groups. **B**) Results of offline changes in sequence-specific learning in OSA and control group: The differences between the low and high frequency triplets indicate sequence-specific learning. There was a decrease in sequence-specific knowledge, such that the learning index of the first epochs of Session 2 was significantly smaller compared to the last epochs of Session 1. There were no significant differences between the OSA and control groups. **C**) The results of offline changes in general skill learning: the differences in overall reaction time between the last epoch of Session 1 and the first epoch of Session 2 regardless of triplet type show general skill learning. There was a trend of improvement in general skill learning. The OSA group showed no offline general skill learning, while the control group showed better performance (smaller RTs) at the beginning of Session 2 compared to the end of Session 1. Error bars indicate SEM.

To investigate learning during Session 1, a mixed design ANOVA was conducted on the first 5 epochs of the data shown in Figure 1A, with TRIPLET (2: high- vs. low-frequency) and EPOCH (5: 1–5) as within-subject factors, and GROUP (OSA vs. control) as a between-subject factor. All significant results are reported together with the *η* ^2^_p_ effect size and Greenhouse Geisser ε correction factors where applicable. Post hoc analyses were conducted by Fisher's LSD pairwise comparisons.

There was significant sequence-specific learning (indicated by the significant main effect of TRIPLET: *F*(1,32) = 15.58, η_p_^2^ = 0.32, *p*<.001), such that RTs were faster on high- than on low-frequency triplets. OSA and control groups showed no differences in sequence-specific learning (TRIPLET×GROUP interaction: *F*(1,32) = 1.61, η_p_^2^ = 0.04, *p* = 0.21).

There was also significant general skill learning (shown by the significant main effect of EPOCH: *F*(4,128) = 28.62, η_p_^2^ = 0.47, *p*<0.001), such that RTs decreased across epochs. OSA and control groups performed at the same level (EPOCH×GROUP interaction: *F*(4,128) = 2.21, η_p_^2^ = 0.06, *p* = 0.12).

The TRIPLET×EPOCH and TRIPLET×EPOCH×GROUP interactions were not significant (*F*(4,128) = 0.94, η_p_^2^ = 0.03 *p* = 0.42; *F*(4,128) = 0.48, η_p_^2^ = 0.01, *p* = 0.69; respectively), indicating that the pattern of learning was similar in the groups. In the overall RT, the OSA group differed significantly from the control group, with slower RTs for the OSA group (main effect of GROUP: *F*(1,32) = 4.95, η_p_^2^ = 0.13, *p* = 0.03). To ensure that this difference in overall RTs did not influence learning measures, we also ran an ANOVA on normalized data (for each participant, the median RTs for high- and low-frequency triplets in each epoch were divided by the overall RT of the first epoch) and found the same results.

### Consolidation of sequence-specific and general skill learning

To investigate the offline changes of sequence-specific and general skill learning we compared the RTs from the last epoch of Session 1 (Epoch 5) and the epoch of Session 2 (Epoch 6) in both groups (for similar analyses see [12], [14]). These variables were submitted to a mixed design ANOVA with TRIPLET (2: high- vs. low-frequency) and EPOCH (2: last epoch of Session 1 and epoch of Session 2) as within-subject factors, and GROUP (OSA vs. control) as a between-subject factor.

The main effect of TRIPLET was significant (*F*(1,32) = 32.34, η_p_^2^ = 0.5, *p*<0.001), thus RTs were faster on high- than low-frequency triplets. It was similar in the OSA and control groups (indicated by the non-significant TRIPLET×GROUP interaction: *F*(1,32) = 1.07, η_p_^2^ = 0.03, *p* = 0.31).

The main effect of EPOCH did not reach significance (*F*(1,32) = 2.34, η_p_^2^ = 0.07, *p* = 0.13) but the EPOCH×GROUP interaction was significant (*F*(1,32) = 9.32, η_p_^2^ = 0.22, *p* = 0.005), suggesting that the OSA and control groups showed significant differences in the offline changes of general skills. The LSD post hoc test revealed that the OSA group showed no offline general skill improvement (*p* = 0.29), while the control group showed better performance (faster RTs) at the beginning of Session 2 compared to the end of Session 1 (*p* = 0.003).

The sequence-specific knowledge did not change significantly during the offline period (TRIPLET×EPOCH interaction: *F*(1,32) = 2.75, η_p_^2^ = 0.08, *p* = 0.11). The OSA and control groups performed on a similar level (TRIPLET×EPOCH×GROUP interaction: *F*(1,32) = 0.29, η_p_^2^ = 0.009, *p* = 0.59). The offline changes of sequence-specific and general skill knowledge are shown on Figure 1B–C, respectively.

There were significant differences in the general RTs between the OSA and control groups, with slower RTs for the OSA group (main effect of GROUP: *F*(1,32) = 6.27, η_p_^2^ = 0.16, *p* = 0.02). ANOVA on normalized data revealed the same results, confirming that the significant difference in offline changes of general skills between the OSA and the control group was not due to general RT differences (EPOCH×GROUP interaction: *F*(1,32) = 11.17, η_p_^2^ = 0.25, *p* = 0.002).

To further confirm the ANOVA results we also analyzed individual differences of sequence-specific and general skill consolidation. In the case of offline sequence-specific changes, we counted the number of participants who exhibited higher sequence-specific learning in Epoch 6 than in Epoch 5 (thus, sequence-specific knowledge in Epoch 6 minus Epoch 5 was above zero, irrespectively of significance testing). A similar number of OSA and control participants (7/17 and 6/17, respectively) showed higher than zero difference in sequence-specific knowledge between Epoch 6 and Epoch 5. Consequently, the number of participants showing the opposite pattern (lower than zero difference between Epoch 6 and Epoch 5) was also similar in the two groups (10/17 and 11/17, respectively). Thus, there was no group difference in sequence-specific consolidation based on this analysis (chi-square(1) = 0.125, p = 0.724) which supports the ANOVA result. In contrast, in the case of general skill consolidation, more controls (14 out of 17) than OSA patients (8 out of 17) showed higher than zero difference in general RTs between Epoch 6 and Epoch 5, thus they were generally faster in Epoch 6 compared to Epoch 5. This group difference in general skill consolidation was significant (chi-square(1) = 4.636, p = 0.031) similarly to the ANOVA result.

## Discussion

Our goal was to investigate the consolidation of non-declarative learning in OSA. We used a relatively complex sequence learning task that allowed us to differentiate between two components of learning: general skill learning and sequence-specific learning. We found differences in offline changes of general skills between OSA patients and controls. The control group showed offline improvement from evening (Learning Phase) to morning (Testing phase), thus, they became faster in the morning after the offline period, while the OSA group did not. In contrast, we failed to find differences in the offline changes of sequence-specific knowledge between the groups. We believe our study to be the first to investigate the consolidation of these two aspects of implicit learning by using a task with complex sequence structures in patients with OSA.

In the Learning Phase the OSA and control group showed similar learning patterns in general skill and sequence-specific learning; however the OSA group demonstrated slower RTs in general. These intact learning curves are in line with previous studies investigating non-declarative learning in this patient population [22], [30], [31]. For example, Nemeth and colleagues [22] and Csabi, Benedek, Janacsek, Katona & Nemeth [45] using the ASRT task also showed intact sequence learning both in children and elderly adult population with sleep-disordered breathing and OSA. In another type of non-declarative memory, Rouleau, Décary, Chicoine & Montplaisir [46] found preserved learning measured by a sensorimotor adaptation task in OSA patients, although a subgroup of them demonstrated deficits in initial learning performance. This subgroup also had difficulties on other neuropsychological tests (e. g. executive functions). Naegelé et al [25] using the same task also found significant but weaker learning in OSA than in the control group. The authors suggest that patients with OSA have difficulties creating new sensorimotor coordination. In sum, these studies suggest that sensorimotor adaptation might be weaker while the less sensorimotor coordination-demanding sequence learning is intact in OSA.

In the overnight consolidation of non-declarative memory we revealed weaker performance on general skill learning in OSA patients compared to the controls who demonstrated offline general skill improvement after the 12-hour delay period. Kloepfer et al [32] found similar results: at the encoding, prior to sleep OSA patients showed similar non-declarative sensorimotor adaptation as the healthy control participants, but they revealed reduced overnight improvement on average RT performance. A recent sequence learning study by Djonlagic et al [16] also demonstrated that OSA patients and controls displayed almost identical performance during the initial learning in the evening, but the control group exhibited significantly more overnight improvement. The authors concluded that this weaker offline performance was caused by sleep fragmentation in OSA.

In the case of sequence-specific learning, we found similar performance between the OSA and control groups not only in online sequence-specific learning but also in the consolidation of sequence-specific knowledge. This result is in line with previous studies that failed to find sleep-related changes in the consolidation of sequence-specific learning in healthy participants [12], [14]. It suggests that sleep might have less influence on this specific aspect of non-declarative learning. This conclusion is also supported by two recent reports. Song & Cohen [47] propose that practice and sleep form different aspects of skill. Their results suggest transition learning (as in the ASRT) to be an implicit component of skills that lacks sleep-dependence. In the other recent consolidation study, Meier and Cock [48] found neither deterioration, nor further improvement in sequence-specific learning over the offline period, however, they found offline improvement in general skill learning.

In conclusion, we demonstrated that the offline changes of two components of implicit sequence learning are differentially affected in OSA: in contrast to the preserved consolidation of sequence-specific knowledge, the consolidation of general skills was weaker compared to the controls. Thus, we suggest that long-term sleep disturbances present in OSA play differential role in these two aspects of consolidation in the case of more complex, probabilistic sequences. Nevertheless, a daytime control condition is needed to investigate whether weaker consolidation of general skills is specific to the actual overnight sleep disturbances or to long-term deficits related to sleep disruption. Our findings underscore the importance of examining more specific and focal cognitive functions in OSA. Creating more sophisticated neuropsychological profiles about the cognitive dysfunctions could not only provide clues about which brain networks may be affected in OSA but also can help develop more effective methods of rehabilitation and treatment.

## References

[1] Krakauer JW, Shadmehr R (2006) Consolidation of motor memory. Trends Neurosci 1: 58–64. 10.1016/j.tins.2005.10.003 PMC2553888 16290273

[2] Axmacher N, Draguhn A, Elger CE, Fell J (2009) Memory processes during sleep: beyond the standard consolidation theory. J Cell Mol Life Sci 66: 2285–2297. 10.1007/s00018-009-0019-1 PMC11115869 19322518

[3] Diekelmann S, Wilhelm I, Born J (2009) The whats and whens of sleep dependent memory consolidation. Sleep Med Rev 13: 309–321. 19251443 10.1016/j.smrv.2008.08.002

[4] Diekelmann S, Born J (2010) The function of sleep. Nat Rev Neurosci 11: 114–126. 20046194 10.1038/nrn2762

[5] Ficca G, Salzauro P (2004) What is sleep for memory? Sleep Med 5: 225–230. 15165527 10.1016/j.sleep.2004.01.018

[6] Fischer S, Nitschke MF, Melchert UH, Erdmann C, Born J (2005) Motor memory consolidation in sleep shapes more effective neuronal representations. J Neurosci 25: 11248–11255. 16339020 10.1523/JNEUROSCI.1743-05.2005 PMC6725908

[7] Gais S, Born J (2004) Declarative memory consolidation: Mechanism acting during human sleep. Learn & Mem 11: 679–685. 10.1101/lm.80504 PMC534696 15576885

[8] Gais J, Lucas B, Born J (2004) Sleep after learning aids memory recall. Learn & Mem 13: 259–262. 10.1101/lm.132106 PMC10807868 16741280

[9] Gais S, Albouy G, Boly M, Dang-Vu TT, Darsaud A, et al (2007) Sleep transforms the cerebral trace of declarative memories. PNAS 104 (47) 18778–18783. 18000060 10.1073/pnas.0705454104 PMC2141853

[10] Fischer S, Hallschmid M, Elsner AL, Born J (2002) Sleep forms memory for finger skills. PNAS 99: 11987–11991. 12193650 10.1073/pnas.182178199 PMC129381

[11] Walker MP, Brakefield T, Morgan A, Hobson JA, Stickgold R (2002) Practice with sleep makes perfect: sleep-dependent motor skill learning. Neuron 35: 205–211. 12123620 10.1016/s0896-6273(02)00746-8

[12] Nemeth D, Janacsek K, Londe Z, Ullman MT, Howard D, et al (2010) Sleep has no critical role in implicit motor sequence learning in young and old adults. Exp Brain Res 201: 351–358. 19795111 10.1007/s00221-009-2024-x

[13] Rickard TC, Cai DJ, Rieth CA, Jones J, Ard MC (2008) Sleep does not enhance motor sequence learning. J Exp Psychol 34: 834–842. 10.1037/0278-7393.34.4.834 18605872

[14] Song S, Howard JH, Howard DV Jr (2007) Sleep does not benefit probabilistic motor sequence learning. J Neurosci 27: 12475–12483. 18003825 10.1523/JNEUROSCI.2062-07.2007 PMC6673329

[15] Urbain C, Schmitz R, Schmidt C, Cleeremans A, Van Bogaert P, et al (2013) Sleep-dependent Neurophysiological Processes in Implicit Sequence Learning. J Cogn Neurosci 25 (11) 2003–2014. 23806174 10.1162/jocn_a_00439

[16] Djonlagic I, Saboisky J, Carusona A, Stickgold R, Malhotra A (2012) Increased sleep fragmentation leads to impaired offline consolidation of motor memories in humans. Plos One 7 (3) e34106. 22470524 10.1371/journal.pone.0034106 PMC3314699

[17] Howard JH, Howard DV Jr (1997) Age Differences in Implicit Learning of Higher Order Dependencies in Serial Patterns. Psychol & Aging 12: 634–656. 10.1037//0882-7974.12.4.634 9416632

[18] Backhaus J, Junghanns K, Born J, Hohaus K, Faasch F, et al (2006) Impaired declarative memory consolidation during sleep in patients with primary insomnia: Influence of sleep architecture and nocturnal cortisol release. Biol Psych 60: 1324–1330. 10.1016/j.biopsych.2006.03.051 16876140

[19] Nissen C, Kloepfer C, Nofzinger EA, Feige B, Voderholzer U, et al (2006) Impaired sleep related memory consolidation in primary insomnia – a pilor study. Sleep 29: 1068–1073. 16944676 10.1093/sleep/29.8.1068

[20] Banno K, Kryger MH (2007) Sleep apnea: clinical investigations in humans. Sleep Med 8: 400–426. 17478121 10.1016/j.sleep.2007.03.003

[21] Archbold KH, Borghesani PR, Mahurin RK, Kapu VK, Landis CA (2007) Neuronal Activation Patterns during Working Memory Tasks and OSA Disease Severity: Preliminary Findings. J Clin Sleep Med 5: 21–27. PMC2637162 19317377

[22] Nemeth D, Csabi E, Janacsek K, Varszegi M, Mari Z (2012) Intact implicit probabilistic sequence learning in obstructive sleep apnea. J Sleep Res 21: 396–401. 22340304 10.1111/j.1365-2869.2011.00983.x

[23] Bedard MA, Montplaisir J, Malo J, Richer F, Rouleau I, et al (1991) Obstructive sleep apnea syndrome: pathogenesis and neuropsychological deficits. J Clin Exp Neuropsychol 13: 950–964. 1779033 10.1080/01688639108405110

[24] Engelman HM, Kingshott RN, Martin SE, Douglas NJ (2000) Cognitive function in sleep apnea/hypopnea syndrome. Sleep 23: 102–108. 10893080

[25] Naegelé B, Thouvard V, Pepin JL, Levy P, Bonnet C, et al (1995) Deficit of cognitive executive functions in patients with sleep apnea syndrome. Sleep 18: 43–52. 7761742

[26] Orth M, Duchna W, Leidag M, Widdig W, Rasche K, et al (2005) Driving simulator and neuropsychological testing in OSAS before and under CPAP therapy. Eur Resp J 26: 898–903. 10.1183/09031936.05.00054704 16264053

[27] Cosentino FII, Bosco P, Drago V, Prestianni G, Lanuzza B, et al (2000) The APOE e4 allele increases the risk of impaired spatial working memory in obstructive sleep apnea. Sleep Med 9: 831–839. 10.1016/j.sleep.2007.10.015 18083630

[28] Pierobon A, Giardini A, Fanfulla F, Callegari S, Majani GA (2008) Multidimensional assessment of obese patients with obstructive sleep apnea syndrome (OSAS): A study of psychological, neuropsychological and clinical relationships in a disabling multifaceted disease. Sleep Med 9 (8) 882–889. 18226950 10.1016/j.sleep.2007.10.017

[29] Macy PM, Kumar R, Woo MA, Valladares BS, Yan-Go FL, et al (2008) Brain Structural changes in obstructive sleep apnea. Sleep 31: 967–977. 18652092 PMC2491498

[30] Lojander J, Kajaste S, Maasilta P, Partinen M (1999) Cognitive function and treatment of obstructive sleep apnea sydrome. J Sleep Res 8: 71–76. 10188139 10.1046/j.1365-2869.1999.00141.x

[31] Wilde MC, Castriotta RJ, Lai JM, Atanasov S, Masel BE, et al (2007) Cognitive impariment in patients with traumatic brain injury and obstuctive sleep apnea. Arch Phys Med Rehab 88: 1284–1288. 10.1016/j.apmr.2007.07.012 17908570

[32] Kloepfer C, Riemann D, Nofzinger EA, Feige B, Unterreiner J, et al (2009) Memory before and after sleep in patients with moderate obstructive sleep apnea. J Clin Sleep Med 5 (6) 540–548. 20465021 PMC2792970

[33] Conway AR, Kane MJ, Bunting M, Hambrick DZ, Wilhelm O, et al (2005) Working memory span tasks: A methodological review and user's guide. Psychol Bull Rev 12: 769–786. 10.3758/bf03196772 16523997

[34] Richardson JT (2007) Measures of short-term memory: a historical review. Cortex 43: 635–650. 17715798 10.1016/s0010-9452(08)70493-3

[35] Daneman M, Blennerhassett A (1984) How to assess the listening comprehension skills of prereaders. J Educ Psychol 76: 1372–1381.

[36] Janacsek K, Tánczos T, Mészáros T, Nemeth D (2009) The Hungarian version of Listening Span task. Hung Rev Psychol 64: 385–406.

[37] Bo J, Jennett S, Seidler R (2011) Working memory capacity correlates with implicit serial reaction time task performance. Exp Brain Res 214: 73–81. 21809082 10.1007/s00221-011-2807-8

[38] Frensch PA, Miner CS (1994) Effects of presentation rate and individual differences in short-term memory capacity on an indirect measure of serial learning. Mem & Cognit 22: 95–110. 10.3758/bf03202765 8035689

[39] Kaufman SB, DeYoung CG, Gray JR, Jiménez L, Brown J, et al (2010) Implicit learning as an ability. Cognition 116: 321–340. 20573341 10.1016/j.cognition.2010.05.011

[40] McGeorge P, Crawford J, Kelly S (1997) The relationships between psychometric intelligence and learning in an explicit and an implicit task. J Exp Psychol 23: 239–245. 10.1037//0278-7393.23.1.239 9028029

[41] Unsworth N, Engle RW (2005) Individual Differences in Working Memory Capacity and Learning: Evidence from Serial Reaction Time Task. Mem & Cognit 33: 213–220. 10.3758/bf03195310 16028576

[42] Janacsek K, Nemeth D (2013) Implicit sequence learning and working memory: correlated or complicated? Cortex 49: 2001–2006. 23541152 10.1016/j.cortex.2013.02.012

[43] Remillard G (2008) Implicit learning of second-, third-, and fourth-order adjacent and nonadjacent sequential dependencies. J Exp Psychol 61: 400–424. 10.1080/17470210701210999 17853201

[44] Howard DV, Howard JH, Japikse K, DiYanni C, Thompson A, et al (2004) Implicit sequence learning: effects of level of structure, adult age, and extended practice. Psychol & Aging 19: 79–92. 10.1037/0882-7974.19.1.79 PMC1224741 15065933

[45] Csabi E, Benedek P, Janacsek K, Katona G, Nemeth D (2013) Sleep disorder in childhood impairs declarative but not non-declarative forms of learning. J Clin Exp Neuropsychol 35: 677–685. 23848557 10.1080/13803395.2013.815693

[46] Rouleau I, Décary A, Chicoine AJ, Montplaisir J (2007) Procedural Skill Learning in Obstructive Sleep Apnea Syndrome. Sleep 25: 398–408. 12071541

[47] Song S, Cohen LG (2014) Practice and sleep form different aspects of skill. Nat Commun 5. 10.1038/ncomms4407 PMC4880362 24647040

[48] Meier B, Cock J (2014) Offline consolidation in implicit sequence learning. Cortex 57: 156–166. 24861420 10.1016/j.cortex.2014.03.009

